# Introducing *Evolution Letters*

**DOI:** 10.1002/evl3.10

**Published:** 2017-05-03

**Authors:** Jon Slate

**Affiliations:** ^1^ Department of Animal and Plant Sciences University of Sheffield Sheffield S10 2TN UK

Welcome to the inaugural issue of *Evolution Letters*. The purpose of this editorial is to explain the journal's scope, main features and policies. Before that though, I will give a brief history of how the journal came into being.

## Background

The idea for a society‐owned evolutionary journal, that featured short articles and a fast time to publication, was first conceived in 2012 by Daphne Fairbairn when she was Editor‐in‐Chief of *Evolution*, the flagship journal of the SSE (Society for the Study of Evolution). Daphne recognized that *Evolution* was at the limit of the number of articles it could publish, and yet there was an increasing need for a top tier journal featuring short papers with high impact, that demanded rapid publication. No journal quite like that existed for evolutionary biologists. An SSE committee, chaired by Charlie Fenster, was formed to look into the feasibility of launching a new journal. At this stage, it became apparent that this was an exciting opportunity to forge closer links between SSE and the European Society for Evolutionary Biology (ESEB). Thus, discussions began around a journal that would be co‐owned by SSE, ESEB, and Wiley‐Blackwell. An oversight committee, chaired by Butch Brodie, was setup. The oversight committee was charged with identifying an editor‐in‐chief, and with ensuring that the journal got off the ground as smoothly as possible. In April 2016, formal agreements were drawn up between the publishers, the societies, and the editor. Following the recruitment of the editorial board, the journal became open to submissions in September 2016.

## A Community Journal

We think there are a number of reasons why *Evolution Letters* is an attractive place to submit your best work. We aim to publish only the highest quality research papers; in particular, we seek exciting papers that demand rapid publication. A “typical” *Evolution Letters* paper is relatively brief, contains exciting new research findings, and is at the cutting‐edge of any area of evolutionary biology. Unfortunately, this means competition for space is tough and quite a high proportion of submissions are not considered for review. These will often be perfectly good manuscripts, but are just not sufficiently exciting to be considered as being among the best in the field. We aim to publish work from across the breadth of evolutionary biology; including not just the areas traditionally covered by our sister journals *Evolution* and *Journal of Evolutionary Biology*, but also topics such as evolutionary medicine, molecular evolution, and paleobiology, which perhaps feature in those journals quite rarely. If you are unsure whether your manuscript falls within the journal's scope, send the editor a presubmission email.

Our Open Access Article Processing Charges (APCs) are modest relative to most other evolutionary biology journals. Furthermore, by publishing in *Evolution Letters*, you are directly supporting other evolutionary biologists. All of the journal's profits that go to SSE and ESEB are put straight back into our community. Both societies fund some great initiatives, with particular support given to young and early career researchers. For example, they fund conferences run for and by graduate students, promote equal opportunities through travel stipends, fund prizes for early career researchers, cover the cost of childcare at the societies’ major meetings, and sponsor various outreach events. By choosing to publish your best work in *Evolution Letters* you are directly helping all of these wonderful initiatives. Our APCs are almost 30% lower for members of SSE and ESEB, so do join one of the two societies if you are considering publishing in *Evolution Letters*. In fact, join them both. There is an additional $50 reduction in article charge to members of both societies, so if you are a member of one, join the other, and the saving will offset the cost of joining the other society.

*Evolution Letters* is entirely open access, so anybody will be able to read your paper when it is published. Many funding bodies will cover the authors’ costs of open access processing charges if the journal is fully Open Access but not if the journal is a hybrid model (i.e., those journals where a library/institution pays subscription fees and authors have the option of paying an APC to make their paper Open Access). We also require authors to make their data available, in common with the other SSE/ESEB journals. *Evolution Letters* is fully integrated with Dryad, and we will not be passing the costs of data deposition on to authors.

## Flexibility

One of the main aims of *Evolution Letters* is to ensure rapid publication of your work. Our current turnaround time from submission to first decision is 25 days, and because we are an online‐only journal, accepted manuscripts can rapidly be published. Articles will appear on Early View as soon as proofs have been received and corrected. Just as importantly though, we are very aware that formatting manuscripts for different journals’ in‐house style can be a time‐consuming and tedious process. We endeavor to make the manuscript submission process as painless as possible. Therefore, we do not require manuscripts to be formatted for *Evolution Letters* until after acceptance. Obviously, it helps if articles are within the intended spirit of the journal though—i.e., relatively succinct, and to the point. This means that if you have had a manuscript turned down by another journal, you can submit the same version of the manuscript you sent to the other journal. Authors may also choose to upload the reviews from the other journal. Doing so may lead to a faster decision time, although we reserve the option of seeking further reviews of the work, especially when the previous reviews were not very comprehensive. We have no policy that says manuscripts cannot be uploaded to bioRxiv or other preprint servers prior to submission to *Evolution Letters*. We will continue to find ways to make the process from manuscript preparation to online publication as rapid, flexible, and author friendly as we can.

## Article Promotion

Of course, once a paper is published, authors will want to see their work receive as much attention as possible. We have an editorial board member, Nicola Hemmings, with the exclusive role of promoting our papers through social media (Twitter handle @EvolLetters) and other routes. Nicola's research expertise is in reproductive biology and sexual selection, and she also has considerable experience in outreach and science communication. Accepted manuscripts all include an “Impact Summary” designed to explain the importance and novelty of the work to somebody less familiar with the field. These summaries are used to help promote the work and should be pitched at a level that a popular science writer, media officer, or an intelligent reader lacking formal training in evolutionary biology would appreciate.

## Our First Articles

Our first articles cover an exciting and diverse set of evolutionary topics. Our very first paper, by Amanda Gibson et al. (2017), is an empirical study examining one of the classical questions in evolutionary biology—the twofold cost of sex. Another paper considers how postcopulatory sexual selection has resulted in transgenerational fitness consequences of sperm storage in an internally fertilizing fish species (Gasparini et al. 2017). Our first macroevolutionary paper (Greenberg and Mooers 2017) uses a comparative approach to show that speciation and extinction rates are correlated in amphibians; a pattern that has not been widely described in other taxonomic groups. A theoretical paper by O'Brien and Wolf (2017) examines how social interactions can drive the evolution of genomic imprinting (parent‐of‐origin expression of genes). The authors conclude that by coordinating gene expression between interacting individuals, genomic imprinting can evolve, because it leads to more successful social interactions between relatives. This “coadaptation theory” leads to testable predictions. Wadgymar *et al*. (2017) is a microevolutionary paper, which describes a very large, replicated common garden experiment on the perennial montane plant *Boechera stricta*. The authors explore both viability and fecundity selection and examine the extent to which locally adapted traits are under selection in a common environment. They also report how viability selection in juveniles can influence the distribution of phenotypes under selection in adults.

We hope you enjoy these papers, and agree that they have set a high benchmark for the journal. Just as importantly though, we hope that you will support *Evolution Letters* and the wider evolutionary biology community by reading the articles we publish and sending your best work to us.
